# Spatio-Temporal Distribution of *Aedes aegypti* (Diptera: Culicidae) Mitochondrial Lineages in Cities with Distinct Dengue Incidence Rates Suggests Complex Population Dynamics of the Dengue Vector in Colombia

**DOI:** 10.1371/journal.pntd.0003553

**Published:** 2015-04-20

**Authors:** Jeiczon Jaimes-Dueñez, Sair Arboleda, Omar Triana-Chávez, Andrés Gómez-Palacio

**Affiliations:** Grupo Biología y Control de Enfermedades Infecciosas—BCEI, Universidad de Antioquia UdeA, Medellín, Colombia; University of Texas Medical Branch, UNITED STATES

## Abstract

**Background:**

*Aedes aegypti* is the primary vector of the four serotypes of dengue virus (DENV1-4), Chikungunya and yellow fever virus to humans. Previous population genetic studies have revealed a particular genetic structure among the vector populations in the Americas that suggests differences in the ability to transmit DENV. In Colombia, despite its high epidemiologic importance, the genetic population structure and the phylogeographic depiction of *Ae. aegypti*, as well as its relationship with the epidemiologic landscapes in cities with heterogeneous incidence levels, remains unknown. We conducted a spatiotemporal analysis with the aim of determining the genetic structure and phylogeography of Colombian populations of *Ae. aegypti* among cities with different eco-epidemiologic characteristics with regard to DENV.

**Methods/Findings:**

Mitochondrial cytochrome oxidase C subunit 1 (COI) - NADH dehydrogenase subunit 4 (ND4) genes were sequenced and analyzed from 341 adult mosquitoes collected during 2012 and 2013 in the Colombian cities of Bello, Riohacha and Villavicencio, which exhibit low, medium and high levels of incidence of DENV, respectively. The results demonstrated a low genetic differentiation over time and a high genetic structure between the cities due to changes in the frequency of two highly supported genetic groups. The phylogeographic analyses indicated that one group (associated with West African populations) was found in all the cities throughout the sampling while the second group (associated with East African populations) was found in all the samples from Bello and in only one sampling from Riohacha. Environmental factors such as the use of chemical insecticides showed a significant correlation with decreasing genetic diversity, indicating that environmental factors affect the population structure of *Ae. aegypti* across time and space in these cities.

**Conclusions:**

Our results suggest that two *Ae. aegypti* lineages are present in Colombia; one that is widespread and related to a West African conspecific, and a second that may have been recently introduced and is related to an East African conspecific. The first lineage can be found in cities showing a high incidence of dengue fever and the use of chemical insecticides, whereas the second is present in cities showing a low incidence of dengue fever where the use of chemical insecticides is not constant. This study helps to improve our knowledge of the population structure of *Ae. aegypti* involved in the diversity of dengue fever epidemiology in Colombia.

## Author Summary

Knowledge on the population structure of *Aedes aegypti*, the main vector of the dengue virus (DENV), is essential to improving dengue fever prevention strategies in endemic countries. In Colombia, despite the epidemiological relevance of dengue fever, the genetic population structure and phylogeography of the vector *Ae. aegypti* is little known. In this study, we evaluated the spatio-temporal structure and phylogeography of Colombian *Ae. aegypti* populations from cities showing different eco-epidemiologic attributes related to dengue fever. Our results indicated that Colombian *Ae. aegypti* populations harbor two mitochondrial lineages related to West and East African ancestors. The lineage related to West African populations is the most frequent and widely distributed in Colombia, and it was found in cities with a high incidence of the dengue fever. A second lineage related to East African populations, which may have been recently introduced in some regions, was found in cities showing a low incidence of dengue. These findings suggest complex population dynamic is involved in dengue fever epidemiology in Colombia, and indicate further studies about biological attributes of the *Ae. aegypti* lineages should be performed.

## Introduction

Dengue is one of the major public health problems in the tropics and is the second-most deadly vector-borne disease in the world after malaria [1]. The mosquito *Aedes aegypti* is the main vector of the four serotypes of dengue flaviviruses (DENV1-4) and yellow fever virus (YFV), and is a known vector of Chikungunya virus [2]. Around the world, approximately 2.5 billion people are at risk of infection with dengue. Moreover, 50 to 100 million new cases of dengue fever (DF) are estimated to occur each year, including up to 500,000 cases of the more severe form of the disease known as dengue hemorrhagic fever (DHF), which has a fatality rate of up to 5% [3]. Thus far, because no effective vaccine is available for DF prevention and no specific drugs are available to treat DF, vector control and entomologic surveillance remain the principal strategies against dengue infection.

Two recognized subspecies of *Ae. aegypti sensu lato (s.l)* have been described according to several molecular and ecological studies [4,5]. The presumed ancient form is *Ae. aegypti formosus (Aaf)*, a sylvan mosquito restricted to the sub-Saharan region of Africa and the *Ae. aegypti aegypti (Aaa)* form referred to as *Ae. aegypti sensu stricto* (*s.s)*, which is widespread across most of the tropical and subtropical regions of the world in association with humans and is considered the main epidemiologically relevant subspecies [6,7]. Recent evolutionary studies based on the molecular analyses of the NADH dehydrogenase subunit 4 (ND4) and cytochrome oxidase I (COI) mitochondrial genes suggest that populations of *Aaa* outside of Africa consist of mosquitoes derived from one of two ancestral clades. One clade is basal and is primarily associated with the mosquito population from Western Africa while the second arises from the first and contains primarily mosquitoes from Eastern Africa [8]. This differentiation is epidemiologically important because certain characteristics such as vector competence for yellow fever and dengue viruses as well as insecticide resistance have been found to vary in populations from different origins [7,9–11].

The sympatric distribution of both *Aaa* clades has been extensively reported in several countries of Central and South America, including Mexico [12], Brazil [13,14], Peru [15], Venezuela [16] and Bolivia [17] where they have been recognized as distinct genetic lineages. Moreover, the presence of only one lineage in some locations is less common and suggests that the total absence of one lineage, or its incomplete colonization, is due to micro-evolutionary forces acting against one lineage to prevent its emergence (i.e., the process of selection to a particular lineage) [13]. In this regard, the seasonal variations in the natural populations of *Aaa* may also explain this occurrence, thereby generating misconceptions about the true absence of a lineage in a particular location; however, clear evidence for this has not emerged thus far. Therefore, the genetic characterization of natural populations over time may help to further elucidate the behavior of these two lineages over time and may explain any possible effects on the epidemiology of dengue.

Colombia is a hyperendemic country for DENV, and the number of cases of DHF and deaths due to dengue have increased dramatically during the last few years, mainly in the Central and Eastern regions [18,19]. For example, in 2010 the number of confirmed deaths due to dengue was 217, the highest reported thus far [19]. Despite the importance of *Aaa* in several cities of Colombia, only two studies were conducted locally in populations in the north and the south of the country, suggesting that the Colombian populations are genetically diverse and are affected by the continued use of chemical insecticides [20,21]. However, many attributes regarding the origin, phylogenetic relationship with previously reported lineages, population structure and population dynamics (i.e., the movement among cities or dispersion among zones having distinct dengue incidence rates) as well as vector competence within populations remain unknown. Knowledge of these biologic attributes is essential to improving mosquito control strategies and in predicting the progressive dispersion and reinvasion across the country. Here we conducted a genetic and phylogeographic analysis using the COI and ND4 mitochondrial genes of 341 *Aaa* individuals from three Colombian cities showing distinct dengue incidence rates that were collected during three samplings during 2012 and 2013. The results indicate two maternal lineages of *Aaa* in Colombia that are differentially distributed across time and space in cities with different eco-epidemiologic characteristics, suggesting distinct colonization events and microclimate variables that modulate the frequency and distribution of each lineage.

## Materials and Methods

### Sample collection and sites of the study

A total of 343 adult mosquitoes were collected in three cities in Colombia between August 2012 and September 2013 with the assistance of the staff of the Vector-Borne Diseases program of the Instituto Nacional de Salud (INS de Colombia). Three samplings were performed in each city, including at least one during the rainy and dry seasons (Table 1). To minimize inbreeding bias in the sample, insect captures using entomologic networks were performed in 20 randomized houses from two neighborhoods separated by more than 1 km and less than 3 specimens from each house were used in the genetic analyses.

**Table 1:** Geographic origin, date and number of Colombian *Aedes aegypti* mosquitoes analyzed in this study.

State	City (code)	Region	Coordinates	Sampling code	Date	Season	Positive houses	N° Samples
Antioquia	Bello (BE)	Andean	6° 20‘ N 75° 35‘ W	A	Sep/2012	Dry	6	38
				B	May/2013	Rain	15	33
				C	Sep/2013	Dry	8	35
				Total			29	106
La Guajira	Riohacha (RI)	Caribbean	11° 31‘ N 72° 55‘ W	A	Aug/2012	Rain	7	43
				B	Dec/2012	Dry	14	36
				C	Apr/2013	Dry	11	42
				Total			32	121
Meta	Villavicencio	Orinoquia	4° 04‘ N 73° 40‘ W	A	Oct/2012	Dry	12	41
	(VI)			B	Apr/2013	Rain	17	35
				C	Jul/2013	Rain	16	39
				Total			45	115
Total							106	343

Notation: A = first, B = second and C = third sampling, respectively.

**Fig 1:**
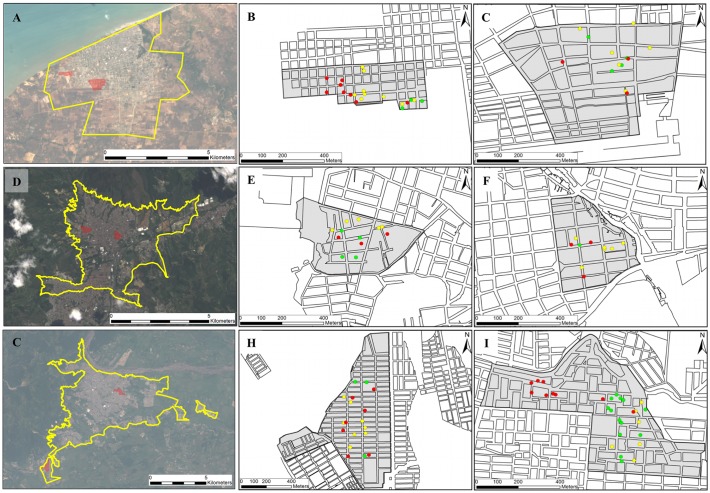
Collection sites of *Ae. aegypti* mosquitoes in Colombian cities of (A) Riohacha, (D) Bello and (G) Villavicencio, and the respective neighborhoods sampled in each city. The surrounding area of each city and the studied neighborhoods (gray area) of Unión (B) and Aeropuerto (C) from RI; Cumbre (E) and Granjas (F) from BE; and Porfia (H) and Popular (I) from VI are shown in the right panel. Green, yellow and red circles indicate the spatial distribution of positive houses for *Ae. aegypti* in the first, second and third sampling, respectively (for details see Table 1).

The cities sampled are localized in three eco-geographic regions with distinct climates and dengue incidence (Fig. 1). Bello (BE) is located in the central Andean region of Colombia in the inter-Andean valleys (Valle de Aburrá) at an altitude of 1250 meters [22] where, because of social control programs and the continuous removal of potential breeding sites of *Ae. aegypti*, the city manifests low incidence rates of between 25.9 to 52.3 cases (per 100,000 inhabitants) and a reduced use of chemical insecticides [19]. Riohacha (RI) is located in northern Colombia’s Caribbean Coast region at an altitude of 2 meters [22], a desert region with a comparatively moderate dengue fever incidence of between 45.3 to 206.8 cases (per 100,000 inhabitants) [19]. Villavicencio (VI) is located in the east in the wide plains of the Orinoquia region at an altitude of 467 meters [22] and is the city that shows the highest incidence rates of dengue in Colombia, between 118.2 to 974.4 cases (per 100,000 inhabitants) [19]. In the last two cities, the strategic plans against dengue fever include entomologic surveillance and the continuous use of chemical insecticides. The geographic origin and the dates of sample collections are detailed in Table 1 and Fig. 1.

### Isolation of mtDNA

To avoid possible Numts amplifications [23–25], and therefore obtaining incorrect phylogenetic and population genetic inferences, enriched mtDNA was obtained for all samples before the PCR amplifications. To achieve this, organelles were obtained according to the method described by Tamura and Aotsuka [26], omitting the alkaline lysis procedure. Each individual adult was homogenized in 500 μl of chilled buffer containing 0.25 M sucrose, 10 mM EDTA and 30 mM Tris-HCl (pH 7.5). The homogenate was centrifuged at 1000 g for 5 min at 4°C and the supernatant (mitochondria, lysosomes and peroxisomes sediment) was retained. The centrifugation process was repeated four times, with the aim of eliminating the greatest amount of nuclear sediment. The resulting supernatant was centrifuged at 12,000 g for 10 min at 4°C and the pellet was dried and stored at -20°C. DNA was extracted from the final pellet using the mosquito standard protocol developed by Collins *et al*. [27].

To test the removal of nuclear DNA, a fragment of variable region D2 of the ribosomal 28S gene was amplified in samples of total DNA of all individuals using the primers D2F 5'- GCGAGTCGTGTTGCTTGATAGTGCAG-3' and D2R 5'-TTGGTCCGTGTTTCAAGACGGG-3' [28]. PCR amplification was performed using a T-1 Thermocycler (Biometra GMBH, D-37079 Goettingen, Germany) with 35 μl reaction mixes/total volume containing 2 μl of 1/16 template DNA dilution, 3.5 μl of 10X reaction buffer (Fermentas), 0.2 mM dNTP, 10 pmol of each primer and 1 UI of Taq polymerase (Fermentas). The amplification reaction was conducted at 95°C for 5 min; 25 cycles at 94°C for 1 min, 50°C for 2 min, 72°C for 2 min; and with a final extension at 72°C for 5 min. PCR products were detected by agarose gel electrophoresis in Tris-borate-EDTA buffer (TBE), stained with GelRed 10,000X diluted 1:10,000 in agarose gel, and visualized under UV light. Only samples showing negative D2-28S amplifications were used for mtDNA amplifications.

### Amplification and sequencing of the ND4 and COI genes

The 400 bp and 900 bp fragments of the ND4 and COI genes, respectively, were amplified individually using primers FOR-ND4 5ʹ—ATTGCCTAAGGCTCATGTAG-3’/REV-ND4 5ʹ-TCGGCTTCCTAGTCGTTCAT-3’ [15], and COI-FOR 5ʹ-GTAATTGTAACAGCTCATGCA-3’/COI-REV 5ʹ-AATGATCATAGAAGGGCTGGAC-3’, respectively [17].

For both genes, PCR amplification was performed using a T-1 Thermocycler (Biometra GMBH, D-37079 Goettingen, Germany) with 50 μl reaction mixes/total volume containing 4 μl of 1/16 template DNA dilution, 5 μl of 10X reaction buffer (Fermentas), 0.2 mM dNTP, 20 pmol of each primer and 1 UI of Taq polymerase (Fermentas). For the ND4 gene, reaction conditions were conducted at 94°C for 5 min, 35 cycles at 94°C for 30 s, 50°C for 30 s and 72°C for 30 s with a final extension at 72°C for 5 min. For the COI gene, 40 cycles were conducted at 94°C for 30 s, 48°C for 30 s and 72°C for 1 min followed by 10 min at 72°C. PCR products were detected by agarose gel electrophoresis in Tris-borate-EDTA buffer (TBE), stained with GelRed 10,000X diluted 1:10,000 in agarose gel, and visualized under UV light. All positive PCR products were purified and sequenced using the Sanger methodology at Macrogen sequencing service, Seoul, South Korea.

### Sequence analyses and genetic differentiation

Sequences were aligned using CLUSTAL W [29] as implemented in BioEdit 7.1.9 [30]. Genetic variability for each marker as well as for the combined COI-ND4 dataset (1178 bp) was evaluated by the number of haplotypes (h), haplotype diversity (Hd) and nucleotide diversity (π) using DnaSP v.5.0 [31]. Significant differences in the π values between different isolated samples were evaluated by analysis of variance (ANOVA) using GraphPad Prism® v.5.1 software. The genetic relationships between individuals were analyzed using a principal coordinate analysis (PCoA) using GenAlEx v.6.4 [32]. The Tajima’s *D* [33] tests was performed to test the hypothesis that all mutations were selectively neutral [34] using DnaSP v.5.0 [31].

Mismatch distribution analysis was performed to identify the pairwise differences spectrum between haplotypes in the different isolates of time and space using DnaSP v.5.0 [31]. A Median-joining haplotype network was built to examine the inter-haplotype relationship among individuals using Network v.4.6.1.1 software [35]. To build the haplotype network, default parameters were used (equal character weight = 10; epsilon value = 10; transversions/transitions weight = 1:1, and connection cost as a criterion).

Genetic differentiation in Colombian *Ae. aegypti* was assessed by Φ_ST_ pairwise comparison, and the genetic structure of isolates in time and space was also assessed using a hierarchical analysis of molecular variance (AMOVA) in GenAlEx v.6.4 [32]. The statistical significance for the population structure analyses was assessed by permutation tests with 1000 iterations.

### Correlation between genetic diversity and environmental variables

Nucleotide (π) and haplotype diversities (Hd) values were compared with the quartiles of relative humidity (HR-Q), *in situ* temperature and number of chemicals interventions with adulticides and larvicides performed in each city during the study using non-parametric Spearman`s correlation coefficients (ρ) with *post-hoc* Holm’s method for multiple comparisons in Hmisc and Rcmdr packages in R software.

### Spatiotemporal analysis of genetic groups

The spatiotemporal distribution of genetic groups was first evaluated through the frequency of individuals in the positive houses. Also, due to any particular spatial arrangement was observed in geographical distribution of *Ae. aegypti* groups, an analysis of Inverse Distance Weighting (IDW) interpolation between the number of positive and negative houses, and the geographic area of each neighborhood studied was performed. The search classes in the IDW interpolation analysis ranked between 0 (absence) to 1 (presence) individuals for each group, with a maximum search area of 2.5° without anisotropy (i.e., circular search area) using ArcGis software (v.9.3; ESRI, Redlands, CA) [36]. This analysis is based on the assumption that the interpolating surface should be influenced most by the nearby points and less by the more distant points [36].

### Phylogeographic analysis

Phylogeographic analysis was performed on the combined COI-ND4 dataset because congruent polymorphism and diversity proportions were observed for separated COI and ND4 genes (S1 Table). The phylogeographic analysis of the Colombian haplotypes included 18 additional haplotypes from Africa, Asia, North America, South America and the Caribbean region (S2 Table). The analysis was executed through a Median-joining haplotype network using Network v.4.6.1.1 software [35] with a preprocessing step to reduce star-shaped haplotypes with a contraction within a radius of five mutational steps. Additionally, a phylogenetic tree including representative most frequent haplotypes (frequency > 0.003) was performed through the neighbor-joining (NJ) method using Mega v.5.1 software [37]. The model parameters HYK + G + I obtained for separated COI and ND4 genes as well as combined COI-ND4 was derived empirically from the best-fitting model estimated from the Akaike information criterion [38,39]. The bootstrap node support was estimated with 1000 replicates and the resulting tree was edited in FigTree v.1.3.1 [40]. The overall topological match score and a well-supported node match score between topologies obtained for separated COI and ND4 genes as well as combined COI-ND4 were calculated using Compare2Trees software [41].

### Accession numbers

All nucleotide sequences are available with GenBank accession codes for COI: KM203140—KM203248 and ND4: KM203249—KM203336.

## Results

### Genetic diversity and differentiation of Colombian *Ae. aegypti*

For the combined COI-ND4 dataset (1178 bp), we identified 160 haplotypes harboring 147 variable sites (12.47%) of which 79 (53.74%) were parsimony informative and 68 (46.25%) were singleton sites (Table 2). The overall Hd was 0.914 ± 0.014 and π was 0.008 ± 0.001, and both haplotype and nucleotide diversities were comparatively higher in BE (0.961 ± 0.013, 0.0113 ± 0.0002) than in RI (0.901 ± 0.026, 0.0044 ± 0.0006) and VI (0.857 ± 0.036, 0.0022 ± 0.0002), respectively (Table 2). As expected for mitochondrial genes showing congruent evolutionary rates with each other, the observed values for Hd and π for both genes were higher in BE than in RI and VI, respectively (S1 Table). A significant difference of π values was observed between the cities, and only RI showed significant differences of these values between samplings (Table 2).

**Table 2:** Summary of genetic diversity and Tajima’s D neutrality tests in Colombian *Ae. aegypti* mosquitoes based on molecular analysis of combined COI-ND4 genes.

City	Smp.	All data combined (n = 298)					group 1 (n = 251)					group 2 (n = 47)				
		n	H	Hd ± SD	π ± SD	D	n	h	Hd ± SD	π ± SD	D	n	h	Hd ± SD	π ± SD	D
BE	A	34	19	0.91 ± 0.03	0.010 ± 1^–3^	1.21	21	9	0.79 ± 0.06	0.004 ± 1^–3^	-1.09	13	10	0.94 ± 0.05	0.004 ± 1^–3^	1.04
	B	22	17	0.96 ± 0.02	0.011 ± 1^–3^	0.95	10	7	0.86 ± 0.10	0.004 ± 1^–3^	-1.86*	12	10	0.97 ± 0.04	0.003 ± 1^–3^	-0.83
	C	33	26	0.98 ± 0.01	0.011 ± 1^–3^	1.26	15	13	0.97 ± 0.03	0.005 ± 1^–3^	-1.01	18	13	0.96 ± 0.03	0.003 ± 1^–3^	-1.39
	Total	89	55	0.96 ± 0.01	0.011 ± 1^–3 RE,VI^	-	46	27	0.88 ± 0.04	0.005 ± 1^–3 VI^	-	43	28	0.96 ± 0.01	0.003 ± 1^–3^	-
RI	A	41	23	0.87 ± 0.04	0.006 ± 1^–3^	-1.45	37	19	0.85 ± 0.05	0.004 ± 1^–3^	-2.12*	4	4	1.00 ± 0.17	0.006 ± 1^–3^	-0.18
	B	32	22	0.92 ± 0.04	0.003 ± 1^–3^	-2.21*	32	22	0.92 ± 0.04	0.003 ± 1^–3^	-2.21*	-	-	-	-	-
	C	40	25	0.90 ± 0.04	0.003 ± 1^–3^	-1.74	40	25	0.90 ± 0.04	0.003 ± 1^–3^	-1.74	-	-	-	-	-
	Total	113	65	0.90 ± 0.02	0.004 ± 1^–3 BE,VI^		109	61	0.89 ± 0.02	0.003 ± 1^–3 VI^		4	4	1.00 ± 0.17	0.006 ± 1^–3^	-
VI	A	37	20	0.85 ± 0.05	0.001 ± 1^–3^	-2.29*	37	20	0.85 ± 0.05	0.001 ± 1^–3^	-2.29*	-	-	-	-	-
	B	28	10	0.59 ± 0.11	0.001 ± 1^–3^	-2.35*	28	10	0.59 ± 0.11	0.001 ± 1^–3^	-2.35*	-	-	-	-	-
	C	31	21	0.95 ± 0.02	0.003 ± 1^–3^	-1.81*	31	21	0.95 ± 0.02	0.003 ± 1^–3^	-1.81*	-	-	-	-	-
	Total	96	49	0.85 ± 0.03	0.002 ± 1^–3 BE,RI^	-	96	49	0.85 ± 0.03	0.002 ± 1^–3 BE,RI^	-	-	-	-	-	
Total	A.B.C	298	160	0.91 ± 0.01	0.007 ± 1^–3^	-	251	128	0.88 ± 0.02	0.003 ± 1^–3^	-	47	32	0.97 ± 0.01	0.004 ± 1^–3^	-

Notation: n = number of samples; h = haplotype number; Hd = haplotype diversity; π = nucleotide diversity; SD = standard deviation; D = Tajima’s D parameter; Smp = sampling; the values for π with a superscript abbreviation represent populations that showed significant differences in nucleotide diversity after performing the one-way ANOVA (*P* < 0.05)

* *P* < 0.05.

**Fig 2:**
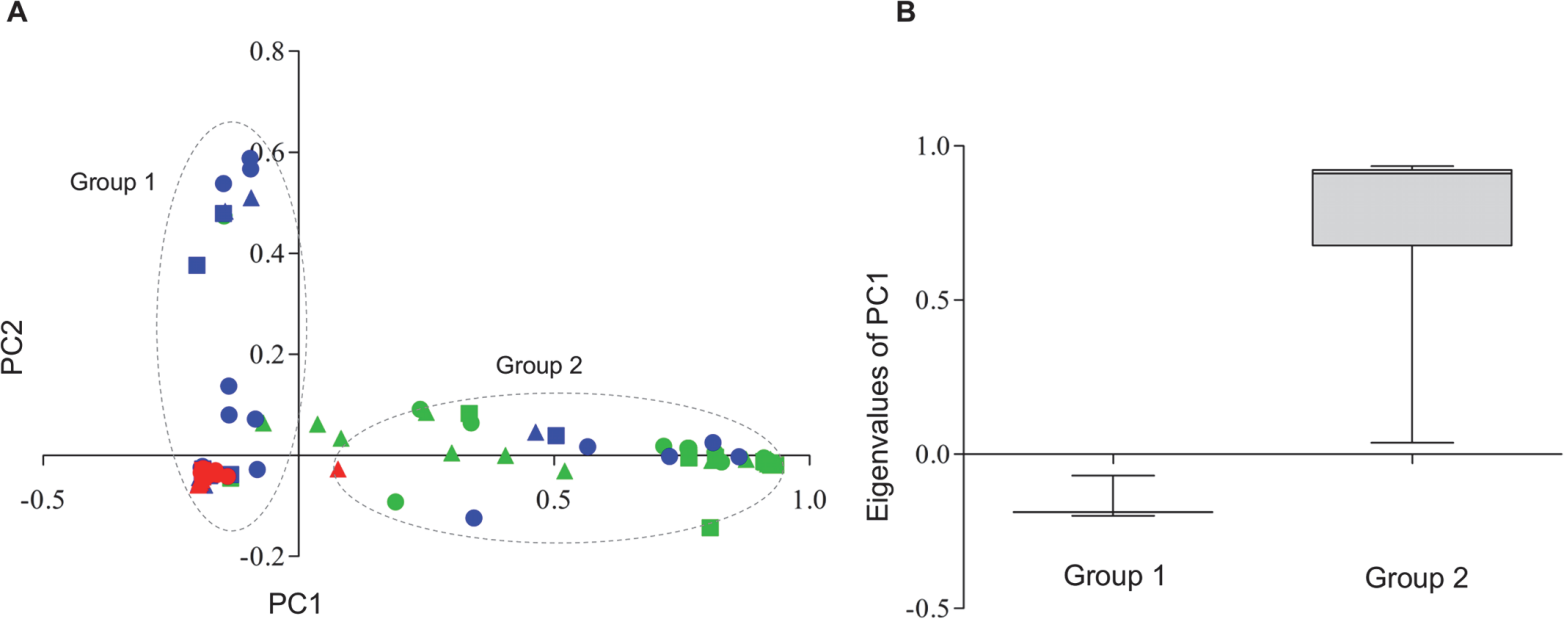
Principal coordinate analysis (PCoA) of combined COI-ND4 genes in *Ae. aegypti* mosquitoes of Colombia. (A) Dotted line represents inferred genetic group 1 and 2 of Colombian mosquitoes projected on the first (x-axis) and second principal coordinates (y-axis), which were derived from a PCoPA analysis. PC1 explains 79.2% of the variance whereas PC2 explains 8%; the color indicates the collection origin: BE (green), RI (blue) and VI (red); the circles, triangles and squares represent A, B, and C samplings, respectively (see Table 1 for details). (B) Box plot of PC1-eigenvalues of group 1 (n = 239) and group 2 (n = 58) derived from PCoA.

**Fig 3:**
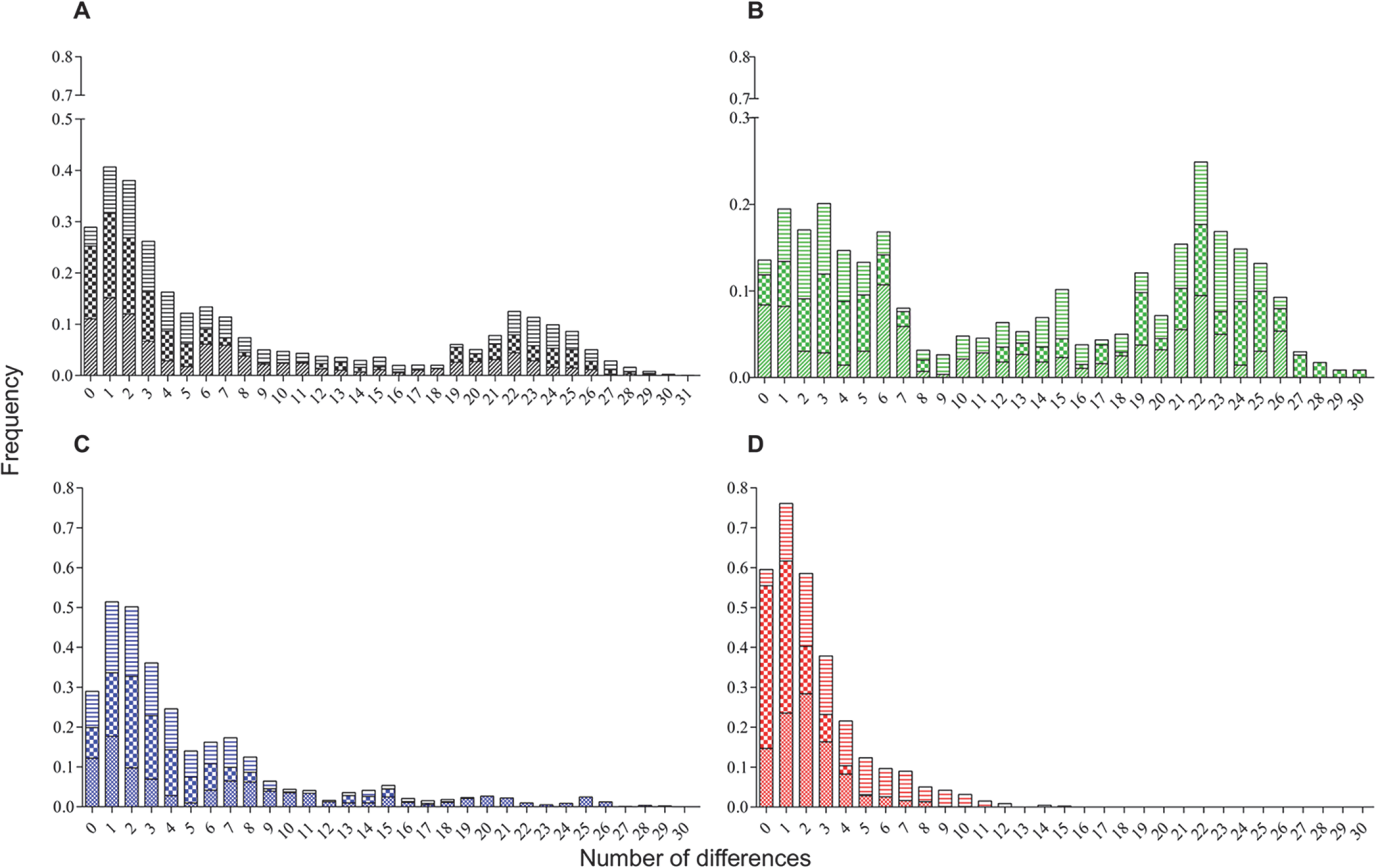
Nucleotide mismatch distribution of combined COI-ND4 genes in *Ae. aegypti* mosquitoes of Colombia. Histograms of mismatch distribution for (A) total haplotypes analyzed and haplotypes within each city of BE (B), RI (C) and VI (D). Color indicates collection origin: BE (green), RI (blue) and VI (red); the cross, bold crossbars and horizontal lines represent the samplings A, B, and C, respectively (see Table 1 for details).

The first two coordinates of the PCoA, harboring 87.2% of the genetic variability in the dataset, roughly indicated that the two groups of haplotypes are inferred (Fig. 2A), as supported by observed PC1-eigen values (Fig. 2B). A first group (group 1) was composed of most haplotypes from all locations and samplings, whereas a more dispersed group (group 2) of haplotypes from BE individuals were collected in all samplings, and a few haplotypes from RI individuals were collected only in the first sampling (sampling A details in Table 1) (Fig. 2A). Nucleotide differentiation between the suggested groups was clearly observed in the mismatch distribution where a bimodal-shaped curve for the entire dataset was observed (Fig. 3A). Furthermore, mismatch distribution performed in each of the cities showed a bimodal trend in BE that harbored haplotypes of insects collected during all samplings (Fig. 3B), whereas in RI and VI a unimodal curve was mostly observed (Fig. 3C, D). For the suggested groups, estimates of genetic diversity (Hd and π) were comparatively lower for group 1 than were observed within group 2 (Table 2). Moreover, only within group 1 were significant differences in π values observed between the cities, with the highest π values occurring in BE and RI compared with VI (Table 2).

**Fig 4:**
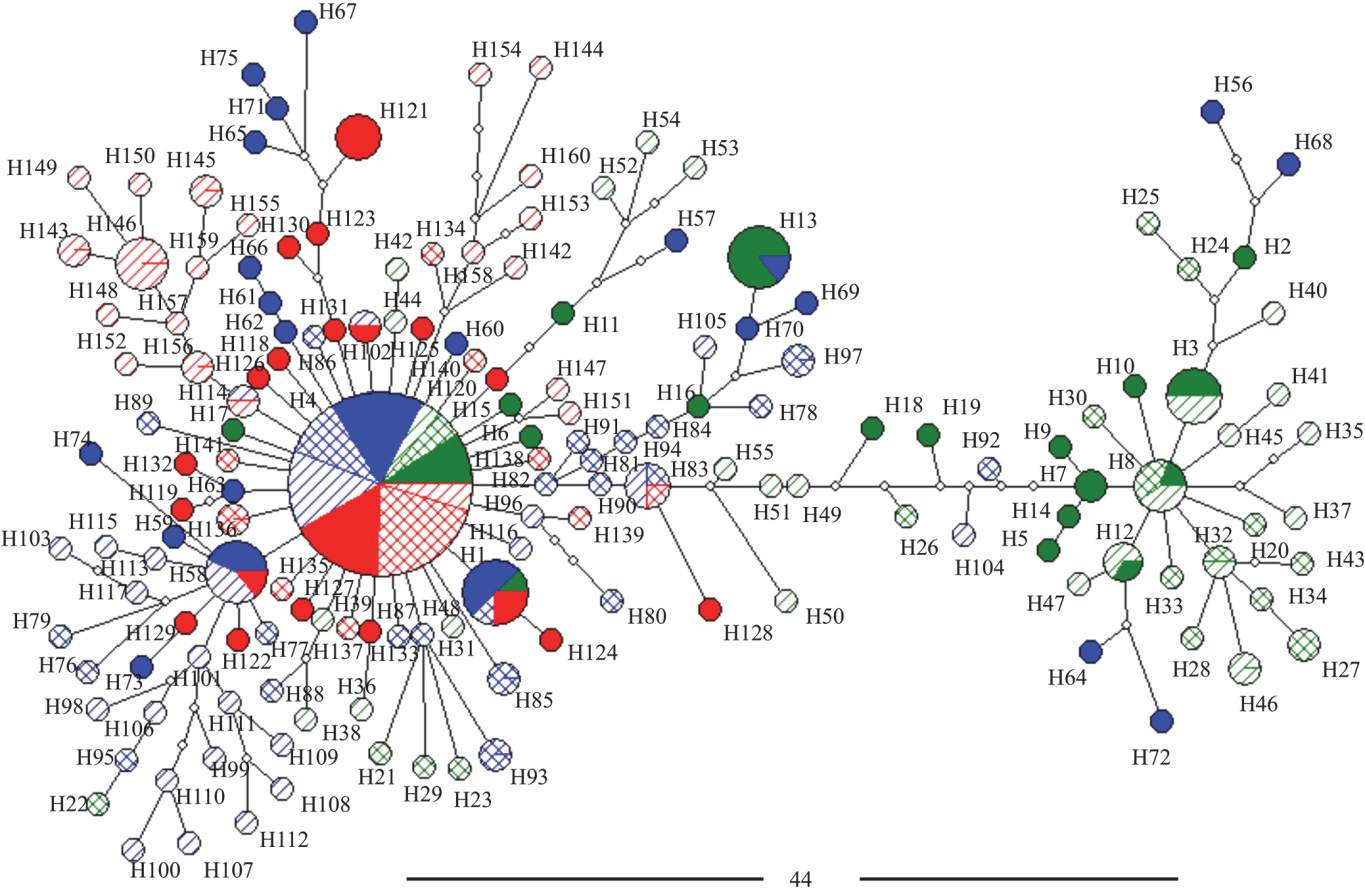
Genealogical relationship among 160 haplotypes of Colombian *Ae. aegypti* based on Median Joining haplotype network of combined COI-ND4 genes. The size of the nodes corresponds to the frequency of the haplotypes; white nodes represent median vectors (hypothetical haplotypes). The black bar represents the number of mutational steps (44 steps) between the nodes of group 1 (left) and group 2 (right). Color indicates the collection origin: BE (green), RI (blue) and VI (red), and respective fill color, crossbars and diagonal lines represent A, B, and C samplings, respectively (see Table 1 for details).

The haplotype network inferred for combined CO1-ND4 (and those obtained for each gene separately, see S1 Fig.) showed a high number of low-frequency haplotypes belonging to two main groups separated by a significant number of mutational steps (Fig. 4). For group 1, a clear star-shaped network was observed, and no apparent differentiation among samples from the cities or samplings was identified. Within this topology, most of the haplotypes found in all of the localities during the three samplings were grouped, and the central and the most frequent haplotype found in all localities/sampling was H4 (frequency = 0.288) (Fig. 4, S3 Table). Moreover, for group 2 a more dispersed haplotype network was observed. Here, only haplotypes from BE plus six haplotypes from RI were grouped (Fig. 4), and the most frequent haplotypes were H3 and H8 (frequencies = 0.020 and 0.016, respectively), which were present only in BE (Fig. 4, S3 Table).

The Tajima’s tests showed negative and significant values (*P* <0.05) in all samplings from VI, and in the second sampling in RI, indicating that an excess of rare haplotypes (i.e., a high number of low-frequency haplotypes) is remained across the studied time in VI and eventually in RI (Table 2). However, for group 1 negative and significant values were obtained in all cities in different samplings, except for group 2, wherein significant values were not observed between cities (Table 2).

Aiming to identify some level of genetic structure among or within the cities as well as among samplings, hierarchical spatiotemporal AMOVA was performed by using HierFstat package in R sofware [42,43]. Hierarchical spatio-temporal AMOVA revealed that the majority of genetic variance was observed among samplings within cities (66%), but having no significant genetic differentiation between them (F_ST_ *=* 0.06). Moreover, similar significant values of genetic differentiation was observed among cities and among samplings (*Fst* = 0.29 and 0.33, respectively), but higher variance component was attributed to among cities (29%) (Table 3). Because those significant spatial and temporal structure observed between cities and samplings may be caused by the presence of the two identified groups in BE and RI, an additional AMOVA was performed comparing the genetic variation and structure between and within groups. The results showed that the highest values of genetic variation could be attributed to comparisons between groups (F_ST_ = 0.79), and between groups within cities (F_ST_ = 0.81) (Table 3). These results indicate that Colombian *Ae. aegypti* genetic differentiation observed is caused by the differential presence of the two genetic groups across of the cities and samplings rather than by any spatio-temporal level.

**Table 3:** Spatio-temporal and genetic hierarchical AMOVA in Colombian *Ae. aegypti* mosquitoes.

Source of variation	F-statistic (*P*-value)	var.component	% variance
Among cities	0.29***	2.88	29.21
Among samplings	0.33***	0.41	4.16
Among samplings within cities	0.06n.s	6.57	66.63
Between groups	0.79***	17.01	79.45
Between groups within cities	0.81***	0.30	1.40
Within groups	0.07***	4.10	19.15

Significance is indicated as ***P<0.001, n.s not significant.

### Genetic diversity and environmental factors of the studied cities

After *post-hoc* Holm`s test, significant (*P* < 0.05) negative Spearman correlation coefficients (ρ) was observed only for nucleotide diversity (π) and number of chemical interventions with adulticides (ρ = -0.73), indicating possible selective pressure is drown in the studied sample (Table 4).

**Table 4:** Spearman`s correlation test for genetic diversities of Colombian *Ae. aegypti* and environmental variables estimated during study in each city.

Independent variable	Spearman`s correlation coefficient (ρ)	
	Nucleotide diversity (π)	Haplotype diversity (Hd)
HR-Q	-0.346	0.051
Temperature (°C)	-0.435	-0.396
No. Interventions with:		
Adulticides	-0.728*	-0.367
Larvicides	-0.055	-0.029

Notation: HR-Q = quartile of relative humidity.

*Significant after Holm’s multiple comparison method (*P* < 0.001).

### Spatial and temporal distribution of genetic groups

**Fig 5:**
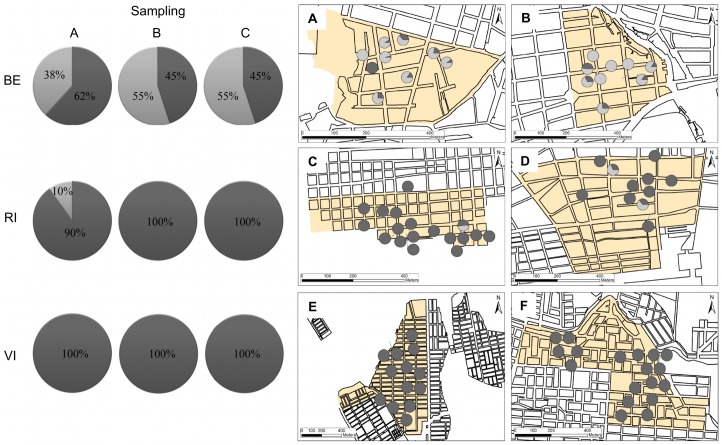
Spatiotemporal distribution of group 1 (dark gray) and group 2 (clear gray) *Ae. aegypti* in the cities studied. Left panel: Temporal distribution of the frequency of group 1 and group 2 of *Ae. aegypti* in each city. Rows represent cities and columns respective samplings A, B and C. Right panel: Spatial distribution of frequency of group 1 and group 2 *Ae. aegypti* across the neighborhoods of Cumbre (A) and Granjas (B) from BE; Unión (C) and Aeropuerto (D) from RI; and Porfia (E) and Popular (F) from VI.

**Fig 6:**
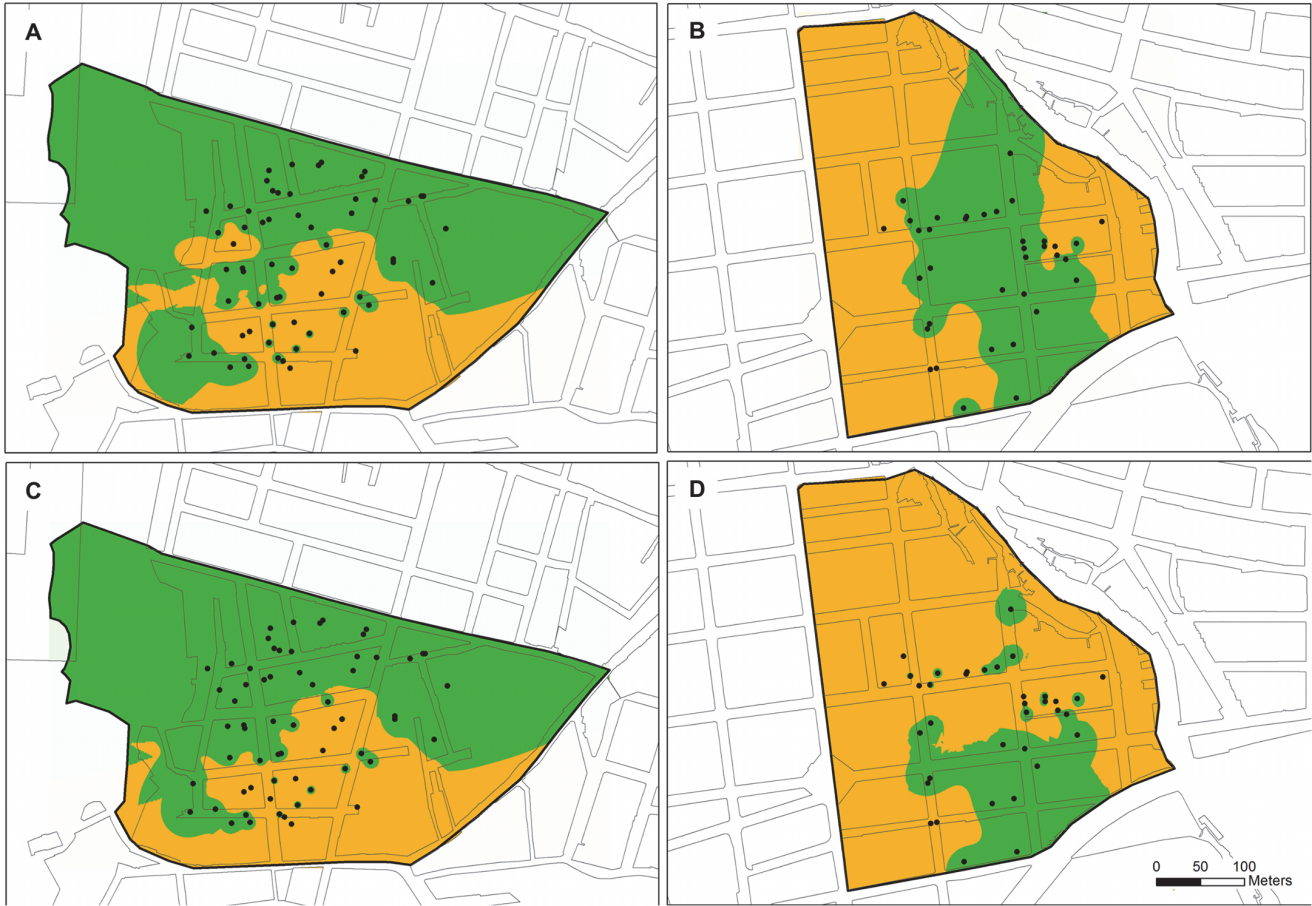
IWD interpolation analysis of group 1 (A, B) and group 2 (C, D) of *Ae. aegypti* in neighborhoods of Cumbre (A, C) and Granjas (B, D) of Bello (BE) city. Green area indicates the classes of search for 0 individuals (absence), and orange areas for 1 or more individuals (presence) in each group. Black circles represent evaluated houses for *Ae. aegypti*.

The spatiotemporal distribution of the frequency of each group across the studied cities was different among them (Fig. 5). Thus, whereas in BE both groups were present with similar frequencies during all samplings, with frequencies ranging from 45%–62% for group 1, and 38%–55% for group 2 (Fig. 5), in RI, group 2 was observed only in sampling A (frequency = 10%) (Fig. 5) and absent in all samplings in VI. Similarly, the distribution of each group in the positive houses indicates that whereas in BE the 66.6% of the positive houses presented both groups, 27.7% were only group 2 and 5.5% were only group 1; in RI, 10.3% of houses presented both groups and in the remaining 89.7% were only group 1; and in VI, 100% of the positive houses showed only group 1 (Fig. 5). Moreover, the interpolation analysis (IWD) performed in the geographic area of neighborhoods from BE indicates that approximately 32.6% of the Cumbre area (Fig. 6A) and 67.3% of Granjas (Fig. 6D) had at least one mosquito of group 1; whereas approximately 32.1% of Cumbre (Fig. 6B) and 80.4% of Granjas (Fig. 6E) had at least one mosquito belonging to group 2. Therefore, a wide potential distribution across the two neighborhoods from BE for both groups was observed, indicating sympatric distribution could be sustained across this city (Fig. 6).

### Phylogeographic relationship of Colombian *Ae. aegypti*

The phylogeographic network build for the entire dataset (COI-ND4) showed two groups of haplotypes that were associated with the haplotypes of America, Asia and Africa belonging to previously suggested mitochondrial lineages I and II [13–17,44] (S2 Fig.). Furthermore, whereas the Colombian haplotypes analyzed here that suggested that group 1 was grouped with haplotypes from West Africa (H1 Cameroon, H1 Guinea and H1 and H3 of Republic of Côte d'Ivoire), and Central (H1 Venezuela) and North America (H1 USA) (reported as lineage I), group 2 was grouped with haplotypes from Eastern Africa (H1 Tanzania), Central America (H1 Mexico), South America (H1 Brazil and H1, H2, H3 and H4 of Bolivia), and the Caribbean region (H2 Martinique) (reported as lineage II), along with the Liverpool strain of *Ae. Aegypti* (S2 Fig.). In the group 1, haplotype 13 (H13) was shared by specimens from Colombia (BE and RI), Venezuela and the USA as haplotype 1 (H1), and in the group 2, the haplotype 3 (H3) of Colombia (BE) was shared with specimens from Bolivia as haplotype (H1) (S2 Fig., S4 Table).

**Fig 7:**
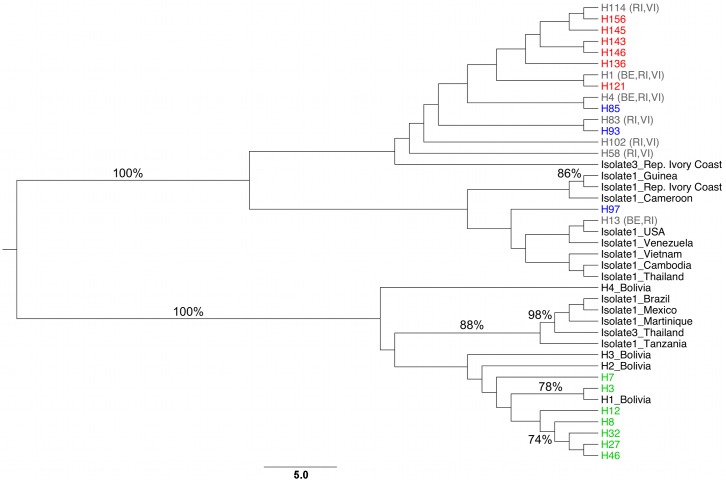
Neighbor-joining (NJ) tree of *Ae. aegypti* haplotypes using HKY + I + G nucleotide substitution model. Green, blue and red taxa indicate haplotypes found exclusively in Colombian cities of BE, RI and VI, respectively. Grey taxa indicates haplotypes found in more than one city (see S4 Table). Node support bootstrap values > 70% are shown on the branch.

In accordance with the phylogeographic network, the phylogenetic tree using the entire dataset (COI-ND4), showed two main monophyletic clades of *Ae. aegypti* supported by a Bootstrap of 100% (Fig. 7). One clade (clade I) included haplotypes that corresponded to the haplotypes suggested here as harboring group 1, whereas the second (clade II) included haplotypes that corresponded to the haplotypes suggested as group 2 (Fig. 7). The overall topological match score between both separated genes (COI and ND4) was of 29.7%, but a high match node score (75%) was observed supporting monophyletic clades corresponding both genetic groups. Similarly, an overall topological match score of 43.4%, and match node score of 87% was observed between COI and combined COI-ND4, and overall topological match score of 51.9%, and match node score of 77.4% for ND4 and combined COI-ND4. These results suggest the suitability of the supermatrix approach supporting *Ae. aegypti* clades reported.

## Discussion

This is the first study of the genetic variation and the molecular phylogeography of *Ae. aegypti* populations of Colombia. Our results reveal a significant level of genetic variation between two previously reported mitochondrial lineages circulating in distinct frequencies across cities with different eco-epidemiologic characteristics during 2012 and 2013. These frequencies, which have fluctuated over the last few years in cities showing distinct dengue incidence rates, indicate that a complex vector-population composition might potentially drive dengue epidemiology in Colombia. However, this specific issue is outside the scope of this work; this hypothesis must be further explored in studies of the association between endogenous factors such as vector competence and insecticide resistance with the distribution of genetic groups in Colombia and dengue fever incidence.

### Genetic traits of Colombian *Ae. aegypti* populations

In this study we observed that values of nucleotide diversity (π) in Colombia are similar to those detected across several Brazilian localities [13,14], Venezuela [45] and Mexico [12] where both lineages have been found, but are comparatively higher than the values detected in 21 localities in Bolivia, where according to the authors, the low genetic diversity in this country is explained by its geographic isolation due to the poor terrestrial access with other populations of the continent [17]. Similar genetic composition between the Colombian and most of the American populations might suggest common processes of reinvasion and gene flow occurring across those countries.

Since the introduction of different genetic groups of *Ae. aegypti* in the same locality may significantly increase the genetic variability of the vector populations [46]. We suggest the high frequency of group 1 and group 2 in BE, the low frequency of group 2 in RI and the absence of this latter group in VI may explain the high genetic diversity observed in BE, followed by RI and VI, respectively as well as genetic structure observed between cities (Table 3). Furthermore, this differentiation may also be related to the different vector control measures implemented in each city because, in BE, the social control campaigns and the elimination of potential breeding sites of *Ae. aegypti* are the main control measures against dengue [19], whereas in RI and VI, the use of insecticide chemicals is constant throughout the year [19]. These findings suggest that in these latter cities, the constant use of insecticides has had a marked effect in the effective size of the mosquito population, as evidenced in the neutrality test and negative correlation between nucleotide diversity and number of chemical interventions with adulticides.

Similar to observations in Brazilian populations [46], higher values of genetic diversity in group 2 than those of group 1, which was the most widespread in the three cities, were obtained for the Colombian *Ae. aegypti* studied. This attribute, as well as the star-shape of the haplotype network exhibited by group 1 suggests that this group is most likely more ancient in Colombia because it presents as central (or basal) and presents the most widespread haplotypes in the country. Additionally, for this group negative and significant values of neutrality testing were observed in the three cities, indicating that it has been subjected to selection pressures across the country. Thus, for example in VI (where only group 1 was found), recent studies demonstrated that mosquito populations have high levels of physiologic resistance to DDT, lambda cyhalothrin and deltamethrin [47], as well as biochemical resistance to organophosphates [48,49]. These findings also suggest that the use of chemical insecticides exerts a strong bottleneck effect on the populations of group 1 in Colombia, although other processes that might reduce the genetic diversity (i.e., founder effects and genetic drift) cannot be discounted [50]. Furthermore, the high frequency of group 2 in BE, where no chemical control is conducted, might suggest that this group is not under strong pressure in this city, contrary to the observations in RI, where this group occurred in lower frequencies and only in one of the samplings.

As relates to the local distribution of *Ae. aegypti* groups, in BE the spatial analyses indicate that both groups are widespread across complete areas in the neighborhoods studied, but group 2 might have higher geographic dispersion (12,6%) compared with group 1 in this city, where the use of chemical insecticides was not reported for sampling periods. These results support the conclusions of some studies, which have shown that mosquito populations with lower levels of resistance to chemical insecticides (in our case, possibly group 2) have high fitness compared to those with a higher degree of resistance (in our case, group 1) without this selection pressure [51]. However, further micro-environmental features that favoring the potential dispersion of group 2 cannot be discarded.

### Environmental factors affecting genetic diversity of Colombian *Ae. aegypti*

*Aedes aegypti* is a poikilothermic species; therefore, changes in response to environmental factors such as humidity, temperature and rainfall can affect the genetic structure of its populations [52,53]. In the present study, it was observed that number of chemical interventions with chemical adulticides was negatively correlated with nucleotide diversity indicating a possible selective process can be drawn in cities where these types of insecticides are used. Several studies have shown that their exacerbated use generates selection pressures on resistant haplotypes, reducing the genetic variability in natural *Ae. aegypti* populations [51,54,55]. We observed this finding in RI and VI, where the number of reported interventions was the highest and the genetic variability was lower compared with BE where the use of chemical insecticides is uncommon. These results, coupled with the high levels of insecticide resistance reported in VI, support the idea that group 1 in this locality is often under environmental pressures that reduce genetic diversity and suggest a possible directional selection of insecticide-resistant haplotypes.

### Phylogeographic picture of Colombian *Ae. aegypti*

In Colombia, *Ae. aegypti* was considered eradicated between 1952 and 1960 as a result of the eradication program initiated in the 1940s by the Pan American Health Organization (PAHO) [56]. Unfortunately, this program was discontinued in 1960, producing a progressive re-invasion of the vector in all regions of the country [56].

Based on the phylogeographic analysis, our data indicate the presence of two genetic groups of *Ae. aegypti*, which is in accordance with mitochondrial lineages previously suggested in the Americas [13–17,44]. These lineages likely evolved from the ancestral population of North Africa and later on dispersed around the world. In this study, the haplotypes belonging to Colombian group 1 exhibited a close relationship with the populations of West Africa (Cameroon, Guinea and the Republic of Côte d’Ivoire) [17], whereas the haplotypes of group 2 were grouped with the populations of East Africa (Tanzania) [17].

Since the work conducted by Tabachnick and Powell [57] in analyzing populations of *Ae. aegypti s.l*. worldwide, it has been suggested that the populations that invaded North America belong to the ancestral clade associated with West African populations, whereas those that invaded South America and the Caribbean region derive from a clade belonging to East African populations [6]. According to this hypothesis, our results indicate that group 1, which might be considered ancestral in Colombia, is related to the West Africa clade. However, group 2, associated with populations of East Africa, may be considered as being recently introduced in some regions of the country (BE and RI) due to the gene flow reported among American populations [50]. A different landscape is observed in the Amazon region of Brazil and Bolivia where the ancestral group is related to East Africa and the West Africa group is less frequent and dispersed [17,46]. These hypotheses indicate that distinct colonization routes might have occurred in northern South America and that multiple introductions of populations derived from ancestral lineages might have occurred in Colombia.

According to the colonization routes of *Ae. aegypti* in the northern regions South America, we suggest that group 1 might have been introduced into Colombia through the land border with Venezuela or by the increase in imports from the USA [50], since H13, belonging to group 1, has been found in BE and RI as well as in Venezuela and USA (in these areas group 1 is termed haplotype 1) [17]. However, it is also possible that the haplotypes of group 1 represent relics of Colombian populations that escaped the DDT-based eradication. Alternatively, the high genetic diversity observed in group 2 suggests that this group was the result of multiple introductions from several American countries such as Bolivia, Mexico or Brazil, as inferred by the close relationship with the haplotypes reported in these countries [17]. These hypotheses indicate that distinct colonization routes might have occurred in northern South America and that multiple introductions of populations derived from ancestral lineages might have occurred in Colombia.

Genetic structure of *Ae aegypti* populations is thought influencing their ability to transmit arbovirus as YFV, Chikungunya and dengue in endemic areas [11]. According to Powell and Tabachinick [58], the vector competence is likely the result of the effects of adaptation for other functions not having anything to do with vector competence (i.e., adaptations accompanying domestication and adaptation of the virus to the mosquito) [58]. Although no vector competence studies have been performed for both genetic lineages so far, it is known that populations of *Ae. aegypti* from West Africa historically are involved in the transmission of YFV, while East Africa population has not been implicated in outbreaks [59]. Based on these features and considering that YFV and dengue virus belong to same family (*Flaviviride*), DENV vectorial capacity of the American mitochondrial genetic groups should be still addressed.

Finally, whereas some works suggest that the presence of two mitochondrial lineages of *Ae. aegypti* is due to a genetically linked faulty production during Numts amplification [24,25], our work confirms that those can be considered as true mtDNA lineages. First, we used a protocol for the extraction of DNA and the amplification of mtDNA that ensured that most of DNA obtained was mitochondrial [26]. Second, the congruent results of both markers used (COI and ND4), showing similar rates of mutation in mtDNA, is contrary to those expected in their respective Numts [24]. Finally, none of the nucleotide sequences of the Colombian haplotypes contained stop codons or insertions/deletions.

### Conclusion

The results demonstrated in this study indicate multiple introductions of *Ae. aegypti* in Colombia, inferring that a distinct ancient origin is involved. Cluster analysis clearly showed two genetic groups, each of which share haplotypes with populations of West Africa and East Africa, respectively. Our results suggest that the group 1 specimens related to West Africa might be present in cities where the use of insecticides is constant, whereas the group 2 specimens related to East Africa are associated with cities exhibiting lower incidence rates, without any selection pressure due to insecticides. The presence of genetically distinct groups in Colombia might imply differences in vector competence for transmitting dengue and urban yellow fever viruses, as well as a differential response to vector control strategies [7,11,60], although this hypothesis must be further supported with more accurate evidence as to how endogenous factors relate with dengue incidence. These findings may contribute to a better understanding of the epidemiologic aspects of dengue fever, and possibly help to improve vector control measures, although the biologic characterization of these lineages remains necessary to conduct a more efficient strategy against dengue fever in Colombia.

## Supporting Information

S1 Table Summary of genetic diversity indices in Colombian *Ae. aegypti* obtained for each of the COI and ND4 genes. (DOC)

S2 Table Geographic origin and GenBank access code of sequences used in the phylogeographic analyses. (DOC)

S3 Table Haplotype frequency and distribution of Colombian *Ae. aegypti* based on the combined COI-ND4 mitochondrial genes. (DOC)

S4 Table Haplotype frequency and distribution of *Ae. aegypti* used in the phylogeographic analysis. (DOC)

S1 Fig Genealogical relationship among haplotypes of Colombian *Ae. aegypti* based on molecular analyses of (a) COI and (b) ND4 genes. The size of the nodes corresponds to the frequency of the haplotypes. The black bar represents the number of mutational steps between the nodes of the suggested groups (see text). Color indicates the collection origin: green BE, blue RI and red VI.
(TIF)

S2 Fig Phylogeographic network of *Ae. aegypti*. Vectors of each Colombian city studied here are represented as fill (BE) green; (RI) blue and (VI) red. South America haplotypes of Bolivia (fill yellow); Brazil (yellow diagonal cross); Venezuela (yellow backward diagonal); North American haplotypes (United States = fill fuchsia, Mexico = fuchsia diagonal cross); Africa (Republic of Ivory Coast = fill orange, Tanzania = orange backward diagonal, Guinea = orange diagonal cross, Cameroon = orange vertical); Asia (Vietnam = fill black, Cambodia = black diagonal cross, Thailand = black backward diagonal); Caribbean region (Martinique = green phosphorescent solid) and the Liverpool strain of *Ae. aegypti* (gray solid) were included in the haplotype network. The haplotypes with (*) are subsets of the haplotypes represented in the S4 Table as obtained by star contraction algorithm (see text).
(TIF)
