# Supplementary material for: Spatio-Temporal Distribution of Aedes aegypti (Diptera: Culicidae) Mitochondrial Lineages in Cities with Distinct Dengue Incidence Rates Suggests Complex Population Dynamics of the Dengue Vector in Colombia
Source: PLoS Negl Trop Dis. 2015 Apr 20;9(4):e0003553. doi: 10.1371/journal.pntd.0003553 (PMC4403987; doi:10.1371/journal.pntd.0003553)
Supplement: S1 Table — (DOC) [file pntd.0003553.s001.doc]

**Suplementary Table S1**. Summary of genetic diversity indices in Colombian *Ae. aegypti* obtained for each COI and ND4 genes.

| Marker (bp) | City | Sampling | N° Samples | Parameter | | | | |
| --- | --- | --- | --- | --- | --- | --- | --- | --- |
|  |  |  |  | h | S | K | Hd ± SD | π ± SD |
| COI (828bp) | BE | A | 34 | 16 | 23 | 6.4 | 0.88 ± 0.03 | 0.007 ± 1^-3^ |
|  |  | B | 25 | 13 | 25 | 7.5 | 0.91 ± 0.03 | 0.009 ± 1^-3^ |
|  |  | C | 35 | 18 | 25 | 7.3 | 0.91 ± 0.02 | 0.008 ± 1^-3^ |
|  |  | Total | 94 | 41 | 47 | 7.3 | 0.92 ± 0.01 | 0.008 ± 1^-3^ |
|  | RI | A | 42 | 18 | 25 | 4.2 | 0.81 ± 0.06 | 0.005 ± 1^-3^ |
|  |  | B | 36 | 14 | 22 | 1.9 | 0.69 ± 0.69 | 0.002 ± 1^-3^ |
|  |  | C | 40 | 13 | 15 | 1.6 | 0.71 ± 0.06 | 0.001 ± 1^-3^ |
|  |  | Total | 118 | 40 | 42 | 2.7 | 0.74 ± 0.04 | 0.003 ± 1^-3^ |
|  | VI | A | 39 | 21 | 23 | 1.9 | 0.84 ± 0.05 | 0.002 ± 1^-3^ |
|  |  | B | 28 | 6 | 7 | 0.5 | 0.38 ± 0.11 | 0.001 ± 1^-3^ |
|  |  | C | 31 | 13 | 13 | 2.1 | 0.83 ± 0.04 | 0.002 ± 1^-3^ |
|  |  | Total | 98 | 39 | 41 | 1.8 | 0.78 ± 0.04 | 0.002 ± 1^-3^ |
|  | Total |  | 310 | 109 | 96 | 4.8 | 0.83 ± 0.02 | 0.005 ± 1^-3^ |
| ND4 (350bp) | BE | A | 38 | 8 | 14 | 5.9 | 0.77 ± 0.04 | 0.016 ± 1^-3^ |
|  |  | B | 33 | 18 | 22 | 6.2 | 0.89 ± 0.04 | 0.017 ± 1^-3^ |
|  |  | C | 33 | 13 | 13 | 5.7 | 0.87 ± 0.03 | 0.016 ± 1^-3^ |
|  |  | Total | 104 | 33 | 29 | 6.0 | 0.85 ± 0.02 | 0.017 ± 1^-3^ |
|  | RI | A | 42 | 17 | 23 | 3.0 | 0.65 ± 0.08 | 0.008 ± 1^-3^ |
|  |  | B | 36 | 15 | 18 | 2.0 | 0.74 ± 0.07 | 0.005 ± 1^-3^ |
|  |  | C | 42 | 15 | 19 | 2.2 | 0.70 ± 0.07 | 0.006 ± 1^-3^ |
|  |  | Total | 120 | 43 | 38 | 2.5 | 0.69 ± 0.04 | 0.007 ± 1^-3^ |
|  | VI | A | 42 | 4 | 5 | 0.2 | 0.18 ± 0.07 | 0.001 ± 1^-3^ |
|  |  | B | 35 | 5 | 6 | 0.3 | 0.26 ± 0.09 | 0.001 ± 1^-3^ |
|  |  | C | 39 | 16 | 25 | 1.9 | 0.81 ± 0.05 | 0.005 ± 1^-3^ |
|  |  | Total | 116 | 23 | 32 | 0.9 | 0.47 ± 0.05 | 0.002 ± 1^-3^ |
|  | Total |  | 340 | 88 | 65 | 4.1 | 0.71 ± 0.02 | 0.011 ± 1^-3^ |

**Notation:** Notation: h = number of haplotypes; S = number of variable sites; K = average number of nucleotide differences; Hd = haplotype diversity; π = nucleotide diversity
