# Supplementary material for: Spatio-Temporal Distribution of Aedes aegypti (Diptera: Culicidae) Mitochondrial Lineages in Cities with Distinct Dengue Incidence Rates Suggests Complex Population Dynamics of the Dengue Vector in Colombia
Source: PLoS Negl Trop Dis. 2015 Apr 20;9(4):e0003553. doi: 10.1371/journal.pntd.0003553 (PMC4403987; doi:10.1371/journal.pntd.0003553)
Supplement: S2 Table — (DOC) [file pntd.0003553.s002.doc]

**Suplementary Table S2.** Geographic origin and GenBank access code of haplotypes used in the phylogeographic analysis.

| Origin | Code | Source | Accession number | |
| --- | --- | --- | --- | --- |
|  |  |  | COI | ND4 |
| Venezuela | Isolate 1 | [18] | [JQ926701.1](http://www.ncbi.nlm.nih.gov/nuccore/JQ926701.1) | JQ926726.1 |
| USA | Isolate 1 | [18] | [JQ926684.1](http://www.ncbi.nlm.nih.gov/nuccore/JQ926684.1) | JQ926725.1 |
| Vietnam | Isolate 1 | [18] | [JQ926685.1](http://www.ncbi.nlm.nih.gov/nuccore/JQ926685.1) | [JQ926723.1](http://www.ncbi.nlm.nih.gov/nuccore/JQ926723.1) |
| Cambodia | Isolate 1 | [18] | [JQ926688.1](http://www.ncbi.nlm.nih.gov/nuccore/JQ926688.1) | [JQ926722.1](http://www.ncbi.nlm.nih.gov/nuccore/JQ926722.1) |
| Thailand | Isolate 3 | [18] | [JQ926692.1](http://www.ncbi.nlm.nih.gov/nuccore/JQ926692.1) | [JQ926721.1](http://www.ncbi.nlm.nih.gov/nuccore/JQ926721.1) |
| Thailand | Isolate 1 | [18] | [JQ926691.1](http://www.ncbi.nlm.nih.gov/nuccore/JQ926691.1) | [JQ926720.1](http://www.ncbi.nlm.nih.gov/nuccore/JQ926720.1) |
| Brazil | Isolate 1 | [18] | [JQ926703.1](http://www.ncbi.nlm.nih.gov/nuccore/JQ926703.1) | [JQ926718.1](http://www.ncbi.nlm.nih.gov/nuccore/JQ926718.1) |
| Guinea | Isolate 1 | [18] | [JQ926700.1](http://www.ncbi.nlm.nih.gov/nuccore/JQ926700.1) | [JQ926717.1](http://www.ncbi.nlm.nih.gov/nuccore/JQ926717.1) |
| Cameroom | Isolate 1 | [18] | [JQ926702.1](http://www.ncbi.nlm.nih.gov/nuccore/JQ926702.1) | [JQ926716.1](http://www.ncbi.nlm.nih.gov/nuccore/JQ926716.1) |
| Tanzania | Isolate 1 | [18] | [JQ926704.1](http://www.ncbi.nlm.nih.gov/nuccore/JQ926704.1) | [JQ926715.1](http://www.ncbi.nlm.nih.gov/nuccore/JQ926715.1) |
| Mexico | Isolate 1 | [18] | [JQ926698.1](http://www.ncbi.nlm.nih.gov/nuccore/JQ926698.1) | [JQ926713.1](http://www.ncbi.nlm.nih.gov/nuccore/JQ926713.1) |
| Martinique | Isolate 2 | [18] | [JQ926697.1](http://www.ncbi.nlm.nih.gov/nuccore/JQ926697.1) | [JQ926712.1](http://www.ncbi.nlm.nih.gov/nuccore/JQ926712.1) |
| Rep. Ivory Coast | Isolate 3 | [18] | [JQ926695.1](http://www.ncbi.nlm.nih.gov/nuccore/JQ926695.1) | [JQ926710.1](http://www.ncbi.nlm.nih.gov/nuccore/JQ926710.1) |
| Rep. Ivory Coast | Isolate 1 | [18] | [JQ926693.1](http://www.ncbi.nlm.nih.gov/nuccore/JQ926693.1) | [JQ926709.1](http://www.ncbi.nlm.nih.gov/nuccore/JQ926709.1) |
| Bolivia | Haplotype 4 | [18] | [JQ926679.1](http://www.ncbi.nlm.nih.gov/nuccore/JQ926679.1) | [JQ926708.1](http://www.ncbi.nlm.nih.gov/nuccore/JQ926708.1) |
| Bolivia | Haplotype 3 | [18] | [JQ926678.1](http://www.ncbi.nlm.nih.gov/nuccore/JQ926678.1) | [JQ926707.1](http://www.ncbi.nlm.nih.gov/nuccore/JQ926707.1) |
| Bolivia | Haplotype 2 | [18] | [JQ926677.1](http://www.ncbi.nlm.nih.gov/nuccore/JQ926677.1) | [JQ926706.1](http://www.ncbi.nlm.nih.gov/nuccore/JQ926706.1) |
| Bolivia | Haplotype 1 | [18] | [JQ926676.1](http://www.ncbi.nlm.nih.gov/nuccore/JQ926676.1) | [JQ926705.1](http://www.ncbi.nlm.nih.gov/nuccore/JQ926705.1) |
|  |  |  |  | |
