# Supplementary material for: Spatio-Temporal Distribution of Aedes aegypti (Diptera: Culicidae) Mitochondrial Lineages in Cities with Distinct Dengue Incidence Rates Suggests Complex Population Dynamics of the Dengue Vector in Colombia
Source: PLoS Negl Trop Dis. 2015 Apr 20;9(4):e0003553. doi: 10.1371/journal.pntd.0003553 (PMC4403987; doi:10.1371/journal.pntd.0003553)
Supplement: S3 Table — (DOC) [file pntd.0003553.s003.doc]

**Supplementary Table S3.** Haplotype frequency and distribution of Colombian *Ae****.*** *aegypti* based on combined COI-ND4 mitochondrial genes.

| City | Sampling | *h* | Name and frequency |
| --- | --- | --- | --- |
|  |  |  |  |
| BE | A | 19 | **H1(0.003)**, H2(0.003), H3(0.010), **H4(0.026)**, H5(0.003), H6(0.003), H7(0.006), H8(0.003), H9(0.003), H10(0.003), H11(0.003), H12(0.003), H13(0.020), H14(0.003), H15(0.003), H16(0.003), H17(0.003), H18(0.003), H19(0.003) |
|  | B | 17 | **H4(0.013)**, H8(0.006), H20(0.003), H21(0.003), H22(0.003), H23(0.003), H24(0.003), H25(0.003), H26(0.003), H27(0.006), H28(0.003), H29(0.003), H30(0.003), H31(0.003), H32(0.003), H33(0.003), H34(0.003) |
|  | C | 26 | H3(0.010), **H4(0.010)**, H8(0.006), H12(0.006), H32(0.003), H35(0.003), H36(0.003), H37(0.003), H38(0.003), H39(0.003), H40(0.003), H41(0.003), H42(0.003), H43(0.003), H44(0.003), H45(0.003), H46(0.006), H47(0.003), H48(0.003), H49(0.003), H50(0.003), H51(0.003), H52(0.003), H53(0.003), H54(0.003), H55(0.003) |
|  | A,B,C | 2 | H4(0.050), H8(0.016) |
| RI | A | 23 | **H1(0.016)**, **H4(0.046)**, H13(0.003), H56(0.003), H57(0.003), H58(0.010), H59(0.003), H60(0.003), H61(0.003), H62(0.003), H63(0.003), H64(0.003), H65(0.003), H66(0.003), H67(0.003), H68(0.003), H69(0.003), H70(0.003), H71(0.003), H72(0.003), H73(0.003), H74(0.003), H75(0.003) |
|  | B | 22 | **H1(0.003)**, **H4(0.030),** H76(0.003), H77(0.003), H78(0.003), H79(0.003), H80(0.003), H81(0.003), H82(0.003), H83(0.003), H84(0.003), H85(0.003), H86(0.003), H87(0.003), H88(0.003), H89(0.003), H90(0.003), H91(0.003), H92(0.003), H93(0.006), H94(0.003), H95(0.003) |
|  | C | 25 | **H4(0.040)**, 58(0.010), H96(0.003), H97(0.006), H98(0.003), H99(0.003), H100(0.003), H101(0.003), H102(0.003), H103(0.003), H104(0.003), H105(0.003), H106(0.003), H107(0.003), H108(0.003), H109(0.003), H110(0.003), H111(0.003), H112(0.003), H113(0.003), H114(0.003), H115(0.003), H116(0.003), H117(0.003) |
|  | A,B,C | 1 | H4(0.117) |
| VI | A | 20 | **H1(0.006)**, **H4(0.046)**, H58(0.003), H102(0.003), H118(0.003), H119(0.003), H120(0.003), H121(0.013), H122(0.003), H123(0.003), H124(0.003), H125(0.003), H126(0.003), H127(0.003), H128(0.003), H129(0.003), H130(0.003), H131(0.003), H132(0.003), H133(0.003) |
|  | B | 10 | **H4(0.060)**, H83(0.003), H134(0.003), H135(0.003), H136(0.006), H137(0.003), H138(0.003), H139(0.003), H140(0.003), H141(0.003) |
|  | C | 21 | **H4(0.013)**, H114(0.003), H142(0.003), H143(0.003), H144(0.003), H145(0.003), H146(0.003), H147(0.003), H148(0.003), H149(0.003), H150(0.003), H151(0.003), H152(0.003), H153(0.003), H154(0.003), H155(0.003), H156(0.003), H157(0.003), H158(0.003), H159(0.003), H160(0.003) |
|  | A,B,C | 1 | H4(0.12) |
| All cities |  | 2 | **H1(0.026), H4 (0.288)** |

**Notation**: (A, B, C) represent the total of first, second and third sampling; h = haplotypes number; number in parenthesis represents the frequency of each haplotype; in bold haplotypes shared among cities in at least one sampling.
