# Supplementary material for: Spatio-Temporal Distribution of Aedes aegypti (Diptera: Culicidae) Mitochondrial Lineages in Cities with Distinct Dengue Incidence Rates Suggests Complex Population Dynamics of the Dengue Vector in Colombia
Source: PLoS Negl Trop Dis. 2015 Apr 20;9(4):e0003553. doi: 10.1371/journal.pntd.0003553 (PMC4403987; doi:10.1371/journal.pntd.0003553)
Supplement: S4 Table — (DOC) [file pntd.0003553.s004.doc]

**Supplementary Table S4**. Haplotype frequency and distribution of *Ae. aegypti* used in phylogeographic analysis.

| Country (city) | h | Name and frequency |
| --- | --- | --- |
| Colombia  (BE) | 52 | H4*[H1(0.003), H4(0.046), H6(0.003), H14(0.003), H16(0.003), H21(0.003), H34(0.003), H36(0.003), H37(0.003), H41(0.003), H45(0.003)], H8*[H5(0.006), H7(0.006), H8(0.018), H9(0.003), H10(0.003), H12(0.009), H19(0.003), H31(0.003), H35(0.003), H38(0.003), H42(0.003)], H15*[H15(0.003)], H29*[H20(0.003), H22(0.003), H27(0.003), H29(0.003)], H32*[H25(0.006), H32(0.003)], H40*[H30(0.006), H40(0.003), H43(0.006), H44(0.003)], H55*[H39(0.003)], H2(0.003), **H3(0.021)**, H11(0.003), **H13(0.018)**, H17(0.003), H18(0.003), H23(0.003), H24(0.003), H26(0.003), H28(0.003), H33(0.003), H46(0.003), H47(0.003), 48(0.003), 49(0.003), 50(0.003), H51(0.003), H52(0.003). |
| Colombia  (RI) | 60 | H4*[H1(0.009), H4(0.115), H57(0.003), H58(0.003), H59(0.003), H75(0.003), H80(0.003), H81(0.003), H82(0.003), H88(0.003), H89(0.003), H90(0.003), H108(0.003)], H15*[H73(0.003), H79(0.003), H91(0.006)], H55*[H55(0.018), H56(0.003), H69(0.003), H72(0.003), 105(0.003), 107(0.003)], H74*[H71(0.003), H74(0.003)], H77*[H76(0.003), H77(0.003), H85(0.003)], H78*[H78(0.009)], H83*[H83(0.003)], H95*[93(0.003), 95(0.003)], H96*[H96(0.003)], **H13 (0.003)**, H53(0.003), H54(0.003), H60(0.003), H61(0.003), H62(0.003), H63(0.003), H64(0.003), H65(0.003), H66(0.003), H67(0.006), H68(0.003), H70(0.003), H84(0.003), H86(0.003), H87(0.006), H92(0.003), 94(0.003), H97(0.003), H98(0.003), H99(0.003), H100(0.003), H101(0.003), H102(0.003), H103(0.003), H104(0.003), H106(0.003), H109(0.003). |
| Colombia  (VI) | 45 | H4*[H1(0.006), H4(0.118), H110(0.003), H112(0.003), H116(0.003), H117(0.003), H118(0.003), H119(0.003), H124(0.003), H125(0.003), H127(0.003), H128(0.006), H129(0.003), H130(0.003), H131(0.003), H132(0.003)], H55*[H55(0.003), H114(0.003), H121(0.003)], H78*[H78(0.003), H120(0.003)], H83*[H145(0.003)], H113*[H111(0.003), H113(0.012)], H137*[H134(0.006), H137(0.015), H140(0.003), H141(0.003)], H138*[H138(0.003), H142(0.003)], H147*[H143(0.003), H147(0.006)], H106(0.003), H115(0.003), H122(0.003), H123(0.003), H126(0.003), H133(0.003), H135(0.003), H136(0.003), H139(0.003), H144(0.003), H146(0.003), H148(0.003), H149(0.003). |
| Venezuela | 1 | **H13(0.003)** |
| USA | 1 | **H13(0.003)** |
| Brazil | 1 | H153(0.003) |
| Mexico | 1 | H158(0.003) |
| Bolivia | 4 | **H3(0.003)**, H150(0.003), H151(0.003), H152(0.003) |
| Martinique | 1 | H157(0.003) |
| Cameroon | 1 | H155(0.003) |
| Guinea | 1 | H156(0.003) |
| Rep. Ivory Coast | 2 | H159(0.003), H96*[H160(0.003)] |
| Tanzania | 1 | H161(0.003) |
| Cambodia | 1 | H162*[154(0.003)] |
| Thailand | 2 | H162*[162(0.003)], H163*[163(0.003)] |
| Vietnam | 1 | H162*[162(0.003)] |
| C. Liverpool | 1 | H163*[164(0.003)] |

**Notation:** h = haplotypes number; the number in parenthesis represents the frequency of each haplotype; in bold the haplotypes shared in Colombia and other countries.
