# Supplementary figures and images for: Spatio-Temporal Distribution of Aedes aegypti (Diptera: Culicidae) Mitochondrial Lineages in Cities with Distinct Dengue Incidence Rates Suggests Complex Population Dynamics of the Dengue Vector in Colombia
Source: PLoS Negl Trop Dis. 2015 Apr 20;9(4):e0003553. doi: 10.1371/journal.pntd.0003553 (PMC4403987; doi:10.1371/journal.pntd.0003553)

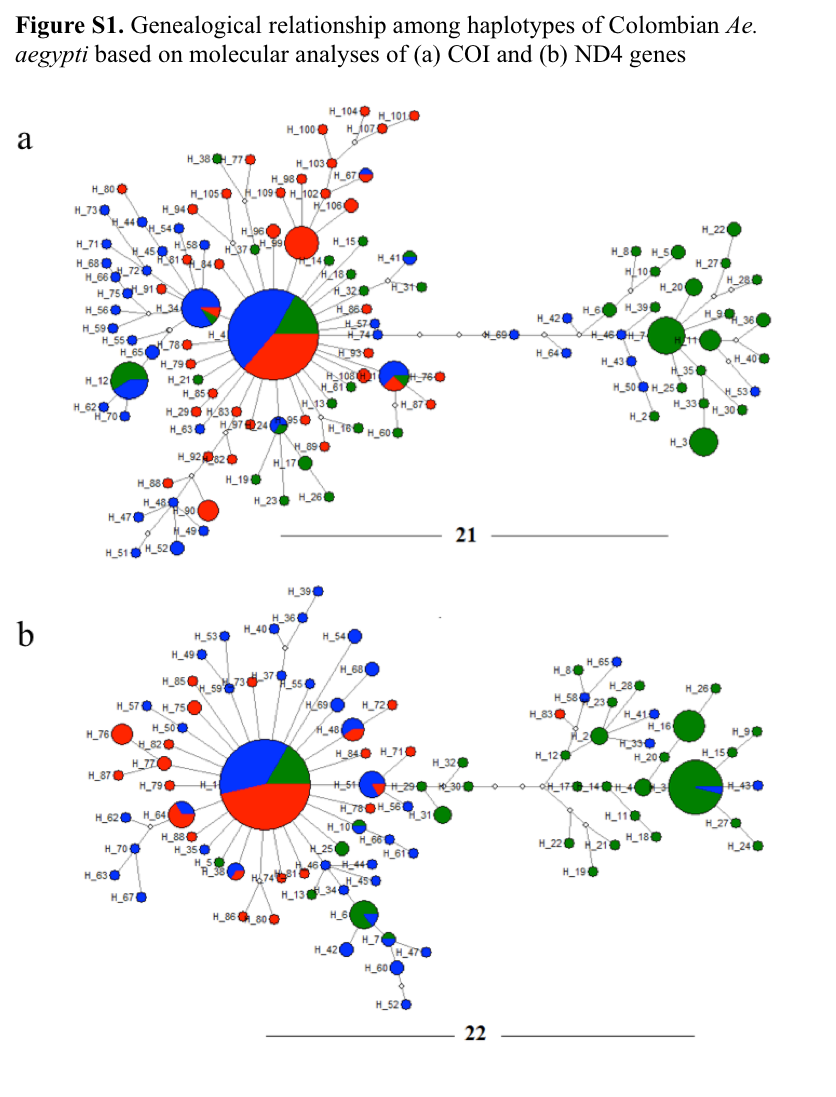

Supplement: S1 Fig — The size of the nodes corresponds to the frequency of the haplotypes. The black bar represents the number of mutational steps between the nodes of the suggested groups (see text). Color indicates the collection origin: green BE, blue RI and red VI. (TIF) [file pntd.0003553.s005.tif]

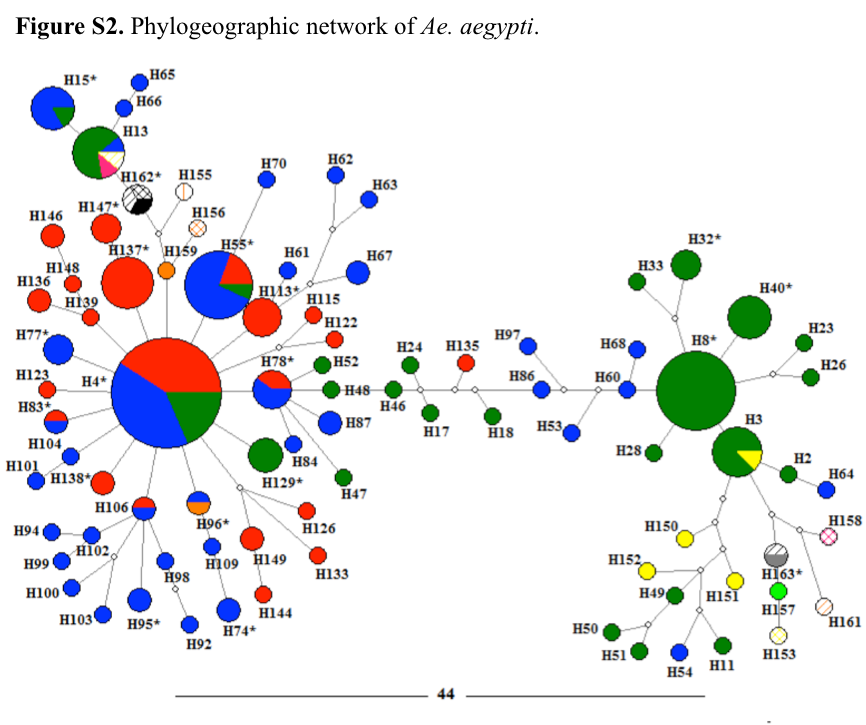

Supplement: S2 Fig — Vectors of each Colombian city studied here are represented as fill (BE) green; (RI) blue and (VI) red. South America haplotypes of Bolivia (fill yellow); Brazil (yellow diagonal cross); Venezuela (yellow backward diagonal); North American haplotypes (United States = fill fuchsia, Mexico = fuchsia diagonal cross); Africa (Republic of Ivory Coast = fill orange, Tanzania = orange backward diagonal, Guinea = orange diagonal cross, Cameroon = orange vertical); Asia (Vietnam = fill black, Cambodia = black diagonal cross, Thailand = black backward diagonal); Caribbean region (Martinique = green phosphorescent solid) and the Liverpool strain of Ae. aegypti (gray solid) were included in the haplotype network. The haplotypes with (*) are subsets of the haplotypes represented in the S4 Table as obtained by star contraction algorithm (see text). (TIF) [file pntd.0003553.s006.tif]
